# Invasion Amid the Shadows: Ecophysiological Dissimilarity and Microhabitat Constraints on an Exotic Succulent in a Mediterranean Ecosystem

**DOI:** 10.1111/ppl.70455

**Published:** 2025-08-15

**Authors:** Erola Fenollosa, Sergi Munné‐Bosch, Marta Pintó‐Marijuan

**Affiliations:** ^1^ Department of Evolutionary Biology, Ecology and Environmental Sciences University of Barcelona Barcelona Spain; ^2^ Institute of Research in Biodiversity (IRBio‐UB) Barcelona Spain

**Keywords:** antioxidants, biological invasions, mediterranean vegetation, *Mesembryanthemum*, photoprotection, seasonal variations

## Abstract

Prevention is the most cost‐effective strategy for managing plant invasions, which includes defining the potential of exotic species to inhabit different environmental conditions. The limiting similarity hypothesis suggests that resemblance to native species facilitates the establishment and spread of exotics in a non‐native range. However, this similarity has rarely been quantified in terms of the physiological strategies used to cope with seasonal environmental variability. Here, we explored for the first time the multivariate ecophysiological similarity between an exotic species and the native community to assess where the invader might succeed. Specifically, we contrasted the physiological annual response of the declared potential invader *Aptenia cordifolia* relative to six coexistent native species in two contrasting environmental conditions (under canopy and at high irradiance) in a Mediterranean‐type ecosystem. The invasive species exhibited distinct physiological responses, demonstrating partial alignment with native traits under specific conditions. At the high irradiance site, the exotic species was the least efficient at counteracting both summer and winter stresses; whereas in the under‐canopy habitat, it exhibited greater ecophysiological dissimilarity from the native community. Our results score the potential of multivariate physiological analysis for guiding habitat prioritization in invasion management and biodiversity conservation in Mediterranean‐type ecosystems.

## 1 Introduction

Invasive species constitute one of the main threats to the conservation of global biodiversity (IPBES 2023). Once an exotic species becomes invasive, its eradication costs increase exponentially; in many cases, this task becomes almost impossible, revealing that prevention may be the only efficient tool against invasive species (Hulme 2007; Munné‐Bosch 2023). Unraveling which mechanisms allow an exotic species to become invasive is essential to prevent future invasions and design cost‐efficient management policies. Despite trait analysis having become an increasingly powerful tool to determine the constraints placed on plant species by the environment, the search for “invasive traits” has yielded contradictory results (Divíšek et al. 2018; Sheppard et al. 2018; Mathakutha et al. 2019). Different traits are known to have differential roles across invasion stages (Renault et al. 2022). The environmental filtering hypothesis states that relatedness to the resident community should facilitate establishment due to similar adaptations to environmental conditions, whereas the limiting similarity or Darwin's naturalization hypothesis states that dissimilarity to native species may benefit alien species spread due to empty niches, reduced competition, or a lack of natural enemies.

Comparing invasive species versus natives to understand invasion success constitutes a useful approach (Domènech and Vilà 2008; Abdallah et al. 2022). Analyzing not only traits but trait complexes or syndromes revealed that invasive species are predominantly different in those traits of coexisting native species (Mathakutha et al. 2019). Despite the recognized importance of species' tolerance to stressful periods in shaping community composition (Valladares et al. 2015), the role of physiological dissimilarity—that is, differences in species' stress‐response strategies or syndromes—in explaining invasion success remains poorly understood. This might be particularly important considering that trait values are not fixed within species (Violle et al. 2012) and that traits strongly depend on environmental conditions (Carmona et al. 2016). Photoprotection and photo‐oxidative stress markers have been proposed as useful tools to unravel invasion success (Fenollosa and Munné‐Bosch 2018; Pintó‐Marijuan and Munné‐Bosch 2013). For instance, their usage revealed a differential photoprotective strategy of the aggressive invader *Carpobrotus edulis* in contrast with a coexistent native to cope with the annual meteorological variations in the Mediterranean Basin (Fenollosa et al. 2017), which could be key to these species' coexistence.

When considering dissimilarities of invasive species in contrast with coexistent natives to understand invasion success, the habitat (vegetation and microhabitat conditions) plays a crucial role (Leishman et al. 2010). Low light availability is an important driving factor controlling morphological and physiological responses of Mediterranean species, independently of water availability (Díaz‐Barradas et al. 2018), that imposes strong photosynthesis limitations (Flexas et al. 2014). The impressive success of the invader *C. edulis* in Mediterranean‐type ecosystems (Munné‐Bosch 2023) relies on its plasticity to show strong ecophysiological performance in terms of growth and photochemical efficiency in different microhabitats differing in light availability (open vs. understory sites), outperforming natives in each community (Traveset et al. 2008). Therefore, in Mediterranean‐type ecosystems, exploring the capacity to cope with environmental conditions in different habitats with different vegetation and light conditions is essential to address the invasive potential of exotic species.

Dissimilarity of exotic species stress response in contrast to the native community has not been tested before as a possible determining factor explaining invasion success. The aim of the study is to test ecophysiological dissimilarity between a declared potential invasive species and coexisting natives in two contrasted environments (with different coexisting species) where the exotic species spreads under high and low irradiance. To achieve this aim, we selected the succulent clonal species *Aptenia cordifolia* (L.f.) Schwantes (= *Mesembryanthemum cordifolium* ), a facultative CAM species (Treichel 1975), native to South Africa and considered invasive in California (Simpson et al. 2023). Despite being already present in the Mediterranean Basin biodiversity hotspot, its invasive potential has not yet been addressed, and it is alerted as a potential invasive species in the area (RD 570/2020). We previously described the potential of *A. cordifolia* to tolerate low water availability regimes (Pintó‐Marijuan et al. 2017), which makes this species a great candidate to expand its presence with climate change, which forecasts increased temperatures and reduced or more variable hydric regimes in the Mediterranean Basin, a climate change hotspot (Tuel and Eltahir 2020; Noto et al. 2023).

## 2 Material and Methods

### 2.1 Experimental Design: Study Sites and Species

**FIGURE 1:**
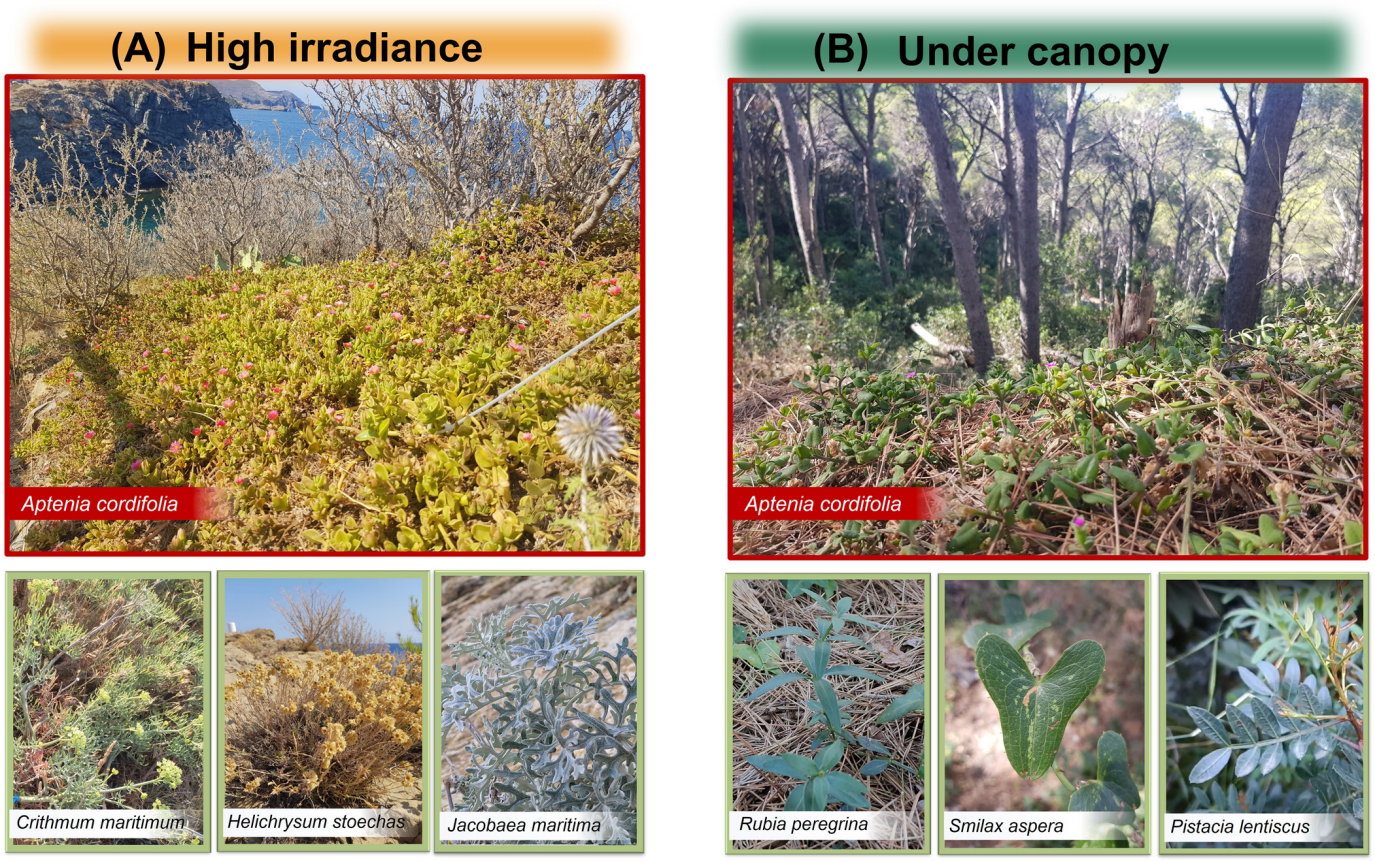
Species selected at the two sites where *Aptenia cordifolia* is present: (A) high irradiance and (B) under canopy. Selected species are described in Supporting Information.

After a prospective study that evaluated the presence and abundance of the potential invasive species *A. cordifolia* (L.f.) Schwantes in multiple locations along the NE Spanish coast, registering also the abundance of coexistent native species, we found that the potential invader occupies a wide range of habitats with different native communities, from rocky coastal habitats, disturbed sandy habitats, to Mediterranean pine forests. Given this preliminary result, we selected two contrasting sites where *A. cordifolia* spreads abundantly. The first site, located in the Alt Empordà region (42°22′14.7″ N, 3°09′57.4″ E) consists of a rocky coastal habitat very close to the Natural Park of Cap de Creus, which includes marine and terrestrial protected areas and constitutes an area of high biological, geological, and landscape quality and diversity with large extensions of woody sclerophyll Mediterranean vegetation. This first site is characterized by a high irradiance area and limited soil depth (entisols) with the habitat type 18a of Rocky cliffs and rocky shores (Catalan vegetation cartography SEMHAVEG, www.ub.edu/geoveg). The second site is located 45 km south, in the Baix Empordà region (41°57′37.0″ N, 3°13′38.9″ E), and is characterized by closed canopies of Mediterranean forest at less than 100 m from the sea. The habitat type of the area is 42aa: Pine groves of Aleppo pine ( *Pinus halepensis* ), with undergrowth of sclerophyll shrubs (gleysols). In each site, from now on “high irradiance” and “under canopy,” respectively, we selected three different abundant and representative native species (see detailed descriptions in Table S1, Figure 1).

**FIGURE 2:**
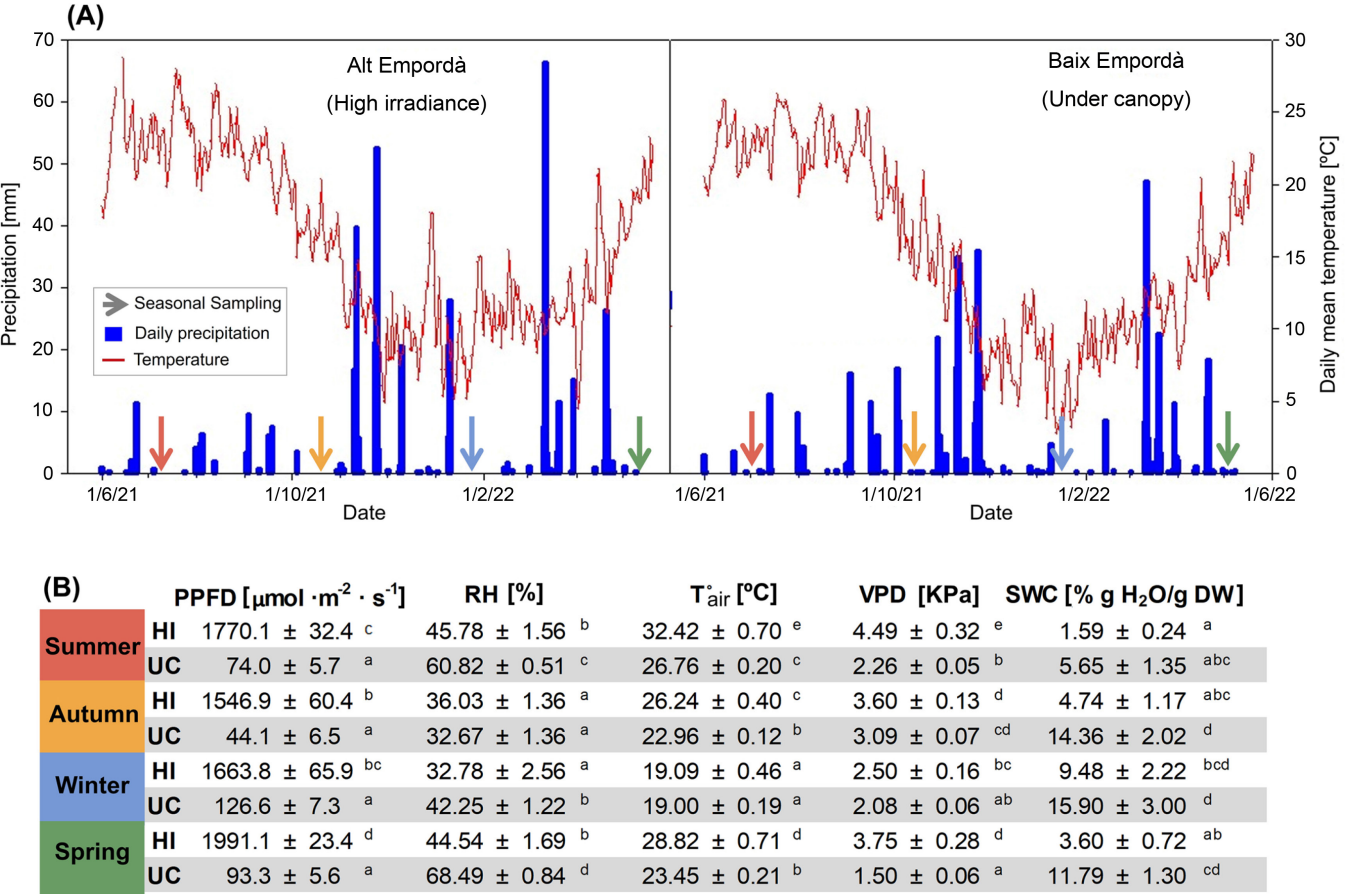
Environmental conditions at the two sites where *Aptenia cordifolia* was found. (A) Precipitation and Daily mean temperature of the two contrasted habitats (high irradiance—HI and under canopy—UC). Arrows point sampling days coloured by each season. (B) Environmental conditions of the sampled days: PPFD: photosynthetic photon flux density, RH: relative humidity, *T*_air_: air temperature, VPD: vapour pressure deficit, SWC: soil water content. Data is represented as mean ± SE (*n* = 8). Different letters correspond to differences between seasons and sites, after Tukey‐b post hoc test in a one‐way ANOVA of the combination of season and site. HI annual mean temperature and annual precipitation is 14.5°C and 600 mm, whereas UC 15.0°C and 650 mm according to the WorldClim Model.

We aimed to capture plant responses to the annual variation of environmental conditions in a Mediterranean climate; therefore, we performed seasonal samplings from June 2021 to April 2022 (Figure 2). To characterize each environmental condition around each individual during samplings in both sites, we analyzed soil water content (SWC), relative humidity (RH), air temperature (*T*_air_) and photosynthetic photon flux density (PPFD). We took eight soil samples across each study site to measure soil water content (SWC) by collecting ca. 10 g of soil, avoiding the top organic matter layer, and sieving the soil with a 2 mm mesh. Soil samples were weighed (soil fresh weight, FWs) and dried at 65°C for a week (soil dry weight, DWs) to calculate SWC [SWC = (FWs − DWs)/DWs]. RH and *T*_air_ were measured using a thermohygrometer (Testo 625, Mediprec) and PPFD was measured at the selected leaf position with an Apogee MQ‐500 Quantum Meter (Apogee Instruments Inc.). We calculated vapor pressure deficit (VPD) as described by Buck (1981).

### 2.2 Ecophysiological Responses: Morphological and Biochemical

Samplings were performed on clear sunny days at midday (Figure 2). We performed measures in situ on each of the eight individuals per species at each site. For each individual, a vertical protractor was used to measure leaf angle, with 180° indicating the tip of the leaf facing the ground and 0° corresponding to the tip of the leaf pointing towards the zenith. In order to estimate leaf temperature, we used a digital laser infrared thermometer (DT‐380, Surpeer). The maximum PSII efficiency (*F*_*v*_/*F*_*m*_) was measured with the fluorometer MINI‐PAM II (Walz), while leaf hydration was calculated as: [*H* = (FW − DW)/DW]; where FW corresponds to the leaf fresh weight and DW to the dry weight, measured as the weight of the leaf after placing it in the drying oven at 60°C until constant weight. Leaf area (LA) was measured at each leaf through image analysis performed with ImageJ Software (NIH) and then the leaf mass per area ratio was calculated (LMA = DW/LA).

Three to five fully developed, non‐senescent leaves were collected, frozen in liquid nitrogen, and stored at −80°C until analyses. One hundred milligrams of completely frozen leaf tissue were powdered with a Tissue Lyser (MM400, Retsch GmbH). 500 μL of methanol containing 0.01% (w/v) butylated hydroxytoluene (BHT) was used as an extraction solvent. After vortexing for 20 s, extraction was performed by using an ultrasound bath (Branson 2510 ultrasonic cleaner, Bransonic) for 20 min, which was followed by 20 s of vortexing again and 10 min of centrifugation at 14,000 g; all at 4°C and avoiding direct light. The supernatant was collected, and the pellet was re‐extracted for two other full extraction cycles. The three resulting supernatants were pooled together, obtaining a final extraction volume of approximately 1.5 mL. This extract was used for the estimation of the extent of lipid peroxidation, total antioxidant capacity, and total phenol contents.

The extent of lipid peroxidation was estimated spectrophotometrically as the accumulation of lipid hydroperoxides (LOOH) following the modified ferrous oxidation‐xylenol orange (FOX) assay as described in Bou et al. (2008). Briefly, two aliquots of the fresh extract were incubated for 30 min in darkness, one marked as positive (+), consisting of a mixture of 150 μL of sample and 150 μL of cold methanol with 0.01% BHT, and the other marked as negative (−), consisting of a mixture of 150 μL of sample and 150 μL of 10 mM triphenylphosphine (TPP). After that, three methodological replicates per sample of 50 μL were incubated for 45 min at room temperature with 150 μL of freshly prepared FOX reagent consisting of 25 mM sulfuric acid, 4 mM BHT, 250 μM ammonium iron (II) sulfate hexahydrate, and 100 μM xylenol orange. Absorbance measurements were made at 560 nm and 800 nm to calculate the LOOH concentration in each sample as [A_560_ − A_800_(+)] − [A_560_ − A_800_(−)], using a H_2_O_2_ calibration curve as a standard.

To quantify the total antioxidant capacity (TAC) of the sample, the 2,2‐diphenyl‐1‐picrylhydrazyl (DPPH) radical scavenging assay was used (Barros 2008). A methanolic dilution of DPPH 0.004% (w/v) was prepared. Each extraction was diluted 1:10 with MeOH, and 100 μL of each dilution was mixed with 1.4 mL of the DPPH reagent. The mix was kept in the dark at room temperature for 10 min; absorbance was measured at 517 nm in a UV–Vis Spectrophotometer (Cecil Aquaris ce7400, Cecil Instruments).

The total phenol content (TPC) was estimated by using the Folin–Ciocalteu reagent assay, following the method described by Singleton and Rossi (1965). Aliquots of 100 μL of 1:10 diluted extracts were mixed with 1 mL of Folin–Ciocalteu. After 4 min, 800 μL of Na_2_CO_3_ (5%, w/v) were added and incubated in the dark for 1 h. The absorbance was measured at 765 nm in a UV–Vis Spectrophotometer (Cecil Aquaris ce7400, Cecil Instruments). The blank was prepared by substituting the same amount of diluted extract with methanol. Two calibration curves for TAC and TPC methods were prepared with gallic acid, and the results were expressed in milligrams equivalents of gallic acid.

### 2.3 Data Analysis

A two‐way ANOVA was fitted to test the significance of the two fixed factors: Species and Season. The Tukey‐b posthoc test was used to test significant differences between species and seasons. Data were tested with Shapiro–Wilk and Levene tests for normality and homoscedasticity and transformed whenever necessary. ANOVA analyses were performed using the SPSS 15.0 statistical package (SPSS Inc.). The significance level was set to *p* < 0.05. In order to test differences in multivariate ecophysiological responses, a multidimensional scaling (MDS) plot was built and significance between species was tested with a PERMANOVA test using the vegan package in R (v. 4.3.1).

## 3 Results

### 3.1 Ecophysiological Adjustments to Annual Climatic Variations

**FIGURE 3:**
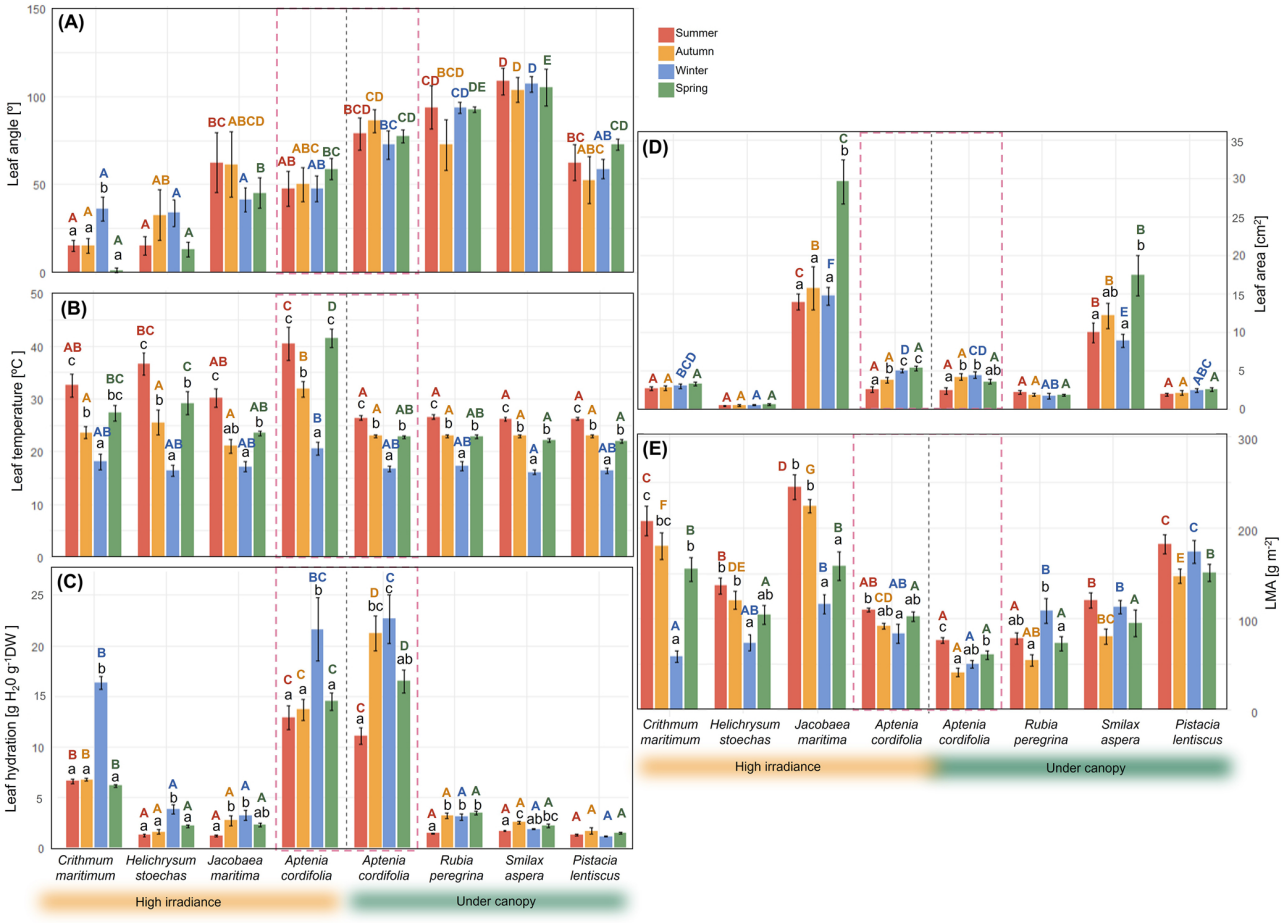
Ecophysiological traits of the multiple coexistent native plants and *Aptenia cordifolia* in two contrasted environments. (A) Leaf angle, (B) leaf temperature, (C) leaf hydration, (D) leaf area, and (E) leaf mass area (LMA). Data represents mean ± SE. Lowercase letters represent significant differences between seasons in each species, whereas capital letters represent significant differences between species in each season (coloured accordingly).

The two contrasted sites where *A. cordifolia* was found showed differences in PFFD, SWC, VPD, and temperature that accompanied changes in the physiology of the species (Figure 2). The high irradiance site showed midday PFFD values from 1500 to 1990 μmol m^−2^ s^−1^, whereas the under‐canopy site maintained a midday PFFD below 130 μmol m^−2^ s^−1^ throughout the study. The maximum sampling air temperature was registered in summer in the high irradiance site, with values of 32.4°C ± 0.7°C, along with the highest VPD (ca. 4.5 kPa). Leaf temperature followed the seasonal oscillations (Figure 3A), with *A. cordifolia* in high irradiance showing the maximum registered leaf temperatures of 40.5°C ± 3.2°C and 41.5°C ± 1.8°C in summer and spring, respectively. Only *Crithmum maritimum* significantly changed the leaf angle across the seasons by increasing the leaf angle in winter, that is, increasing solar exposure by orienting the tip of the leaf more horizontally (Figure 3A). This native species, together with *Helichrysum stoechas*, showed the smallest leaf angles, below 45° (i.e., with the tip of almost vertical). Generally, species in the high irradiance site maintained lower leaf angles than species growing under the canopy and maintained leaf angles around 90° (i.e., horizontal leaf; Figure 3A).

We observed two different patterns in native species seasonal LMA variations: whereas under‐canopy species showed minimum LMA in autumn and spring, species from the high‐irradiance site showed minimum LMA values in winter (with the exception of *H. stoechas*, that maintained LMA stable across seasons; Figure 3E). The potential invasive species *A. cordifolia* showed the highest leaf hydration values, around 16.76 ± 0.78 gH_2_O g^−1^ DW, with the highest values found during winter in both sites. This pattern of maximum leaf hydration in winter was also observed in native species growing in the high irradiance site. Native species in the under‐canopy site showed minimum leaf hydrations in summer and recovered for the following seasons, or, as in the case of *Pistacia lentiscus* , it showed no seasonal changes in leaf hydration (Figure 3C).

### 3.2 Photoinhibition and Biochemical Responses to Mediterranean Stress

**FIGURE 4:**
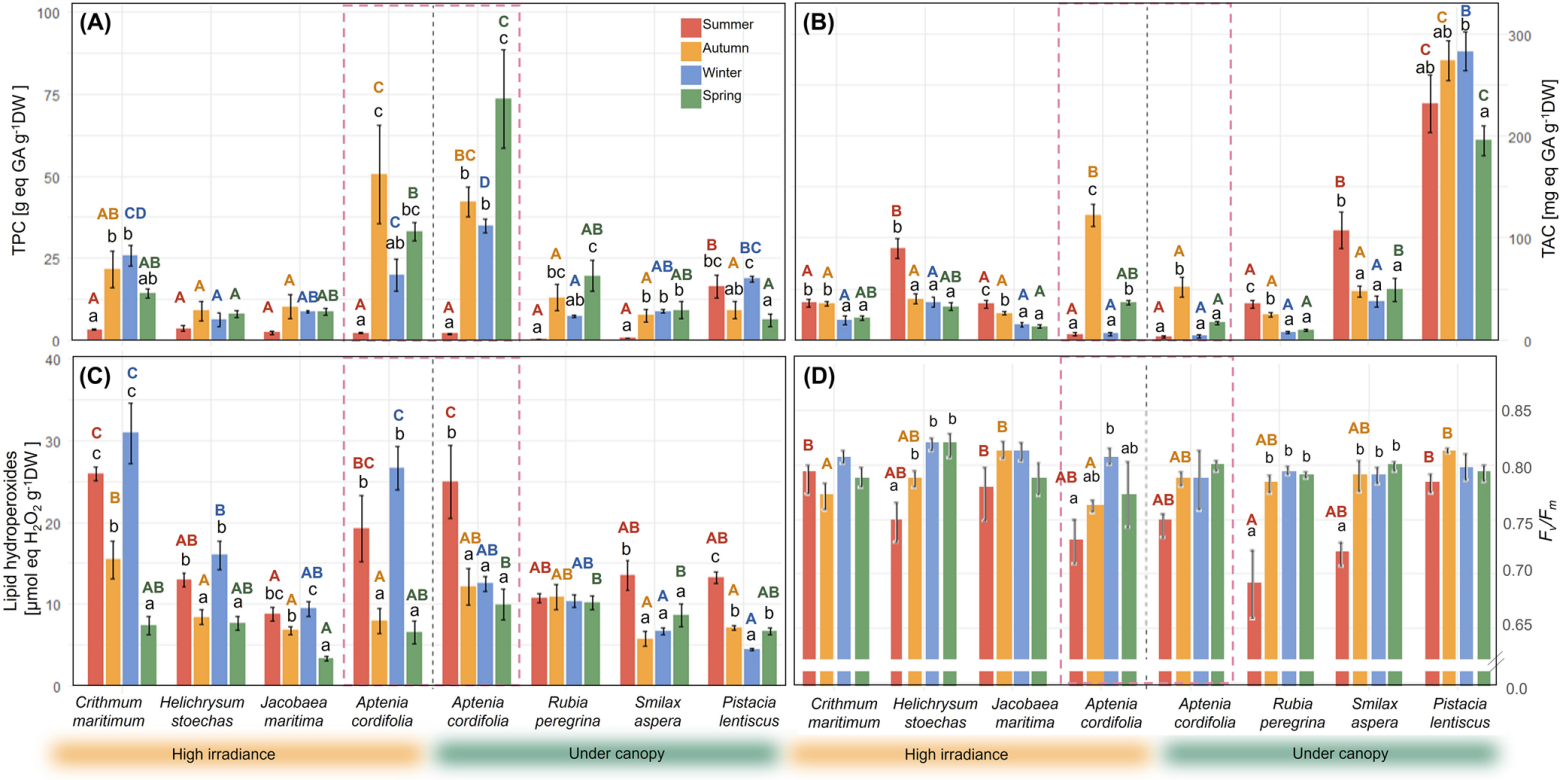
Antioxidant annual variation and photoinhibition of the multiple coexistent native plants and *Aptenia cordifolia* in two contrasted environments. (A) total phenol content (TPC), (B) total antioxidant capacity (TAC), (C) lipid hydroperoxides and (D) maximum efficiency of PSII (*F*_*v*_/*F*_*m*_). Data is represented as mean ± SE (*n* = 8). Lowercase letters represent significant differences between seasons in each species, whereas capital letters represent significant differences between species in each season (coloured accordingly).

*A. cordifolia* showed the highest TPC among all other species but showed strong seasonal variations, with almost 10 times less TPC during summer (Figure 4A). However, almost all other species followed a similar pattern of minimum TPC during summer, with the exception of *P. lentiscus* , which showed the highest TPC summer values and the overall highest TAC values (Figure 4A,B). The rest of the native species showed their TAC maximum levels in summer, while the exotic species showed its maximum in autumn in both sites (Figure 4B). The accumulation of lipid hydroperoxides showed two distinctive seasonal patterns between all species growing in the high‐irradiance site and under‐canopy (Figure 4C). Whereas species growing in the high‐irradiance site showed high levels of LOOH in both summer and winter, species growing under the canopy showed their higher seasonal levels in summer (Figure 4C). This is especially clear in *A. cordifolia* , which only showed this double lipid hydroperoxides peak when growing under high irradiance (Figure 4C). During summer, *Rubia peregrina* , *Smilax aspera* (native species living in the UC site) and *A. cordifolia* growing under high irradiance showed transient photoinhibition with *F*_*v*_/*F*_*m*_ values under 0.75 (Figure 4D).

### 3.3 Seasonal Physiological Dissimilarity Between Species

**FIGURE 5:**
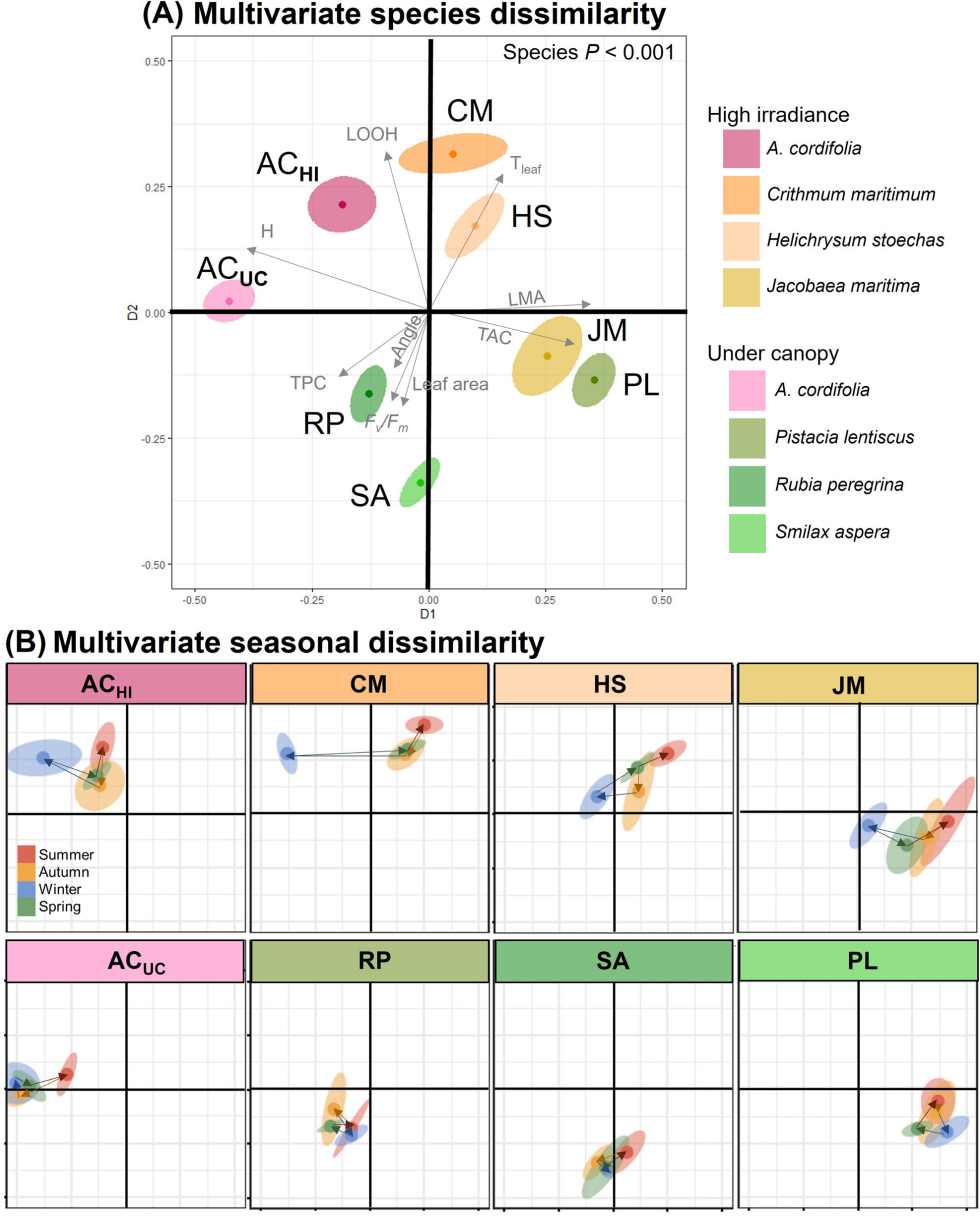
Multivariate ecophysiological dissimilarity between the exotic and coexistent native plants. (A) Multidimensional physiological annual responses of coexistent native plants and *Aptenia cordifolia* in two contrasted environments (HI: high irradiance, UC: under canopy). The left plot corresponds to the multidimensional scaling analysis (MDS) considering all measured traits in all seasons. Ellipses constitute 95% confidence intervals, with their centroid. Grey arrows indicate the direction and strength of the different traits explaining global variability. (B) Species isolated within the MDS and their multidimensional seasonal response. Coloured arrows guide the seasons march. Species traits and full names can be found in Tables S1 and S2 respectively.

The multidimensional analysis considering physiological traits showed an ecophysiological differentiation between species (*p* < 0.001; Figure 5A). Under‐canopy native species were placed at lower values of the *Y*‐axis, thus having in common a higher leaf area, leaf angle, *F*_*v*_/*F*_*m*_, and TPC, while showing lower leaf temperatures and LOOH. Native species from the high irradiance site showed a similar dispersion, with *H. stoechas* and *C. maritimum* being more similar than *Jacobaea maritima* , which shared the same physiological strategy with the under‐canopy site species *P. lentiscus* , with high TAC and LMA values. In general, *H. stoechas* and *C. maritimum* showed an overall higher leaf angle, lipid hydroperoxides, and smaller leaves. Interestingly, *A. cordifolia* from the two sites showed non‐overlapping 95% confidence intervals, revealing significant differentiation of the physiological strategy at both sites. *A. cordifolia* growing in the high irradiance area showed closer performance to *C. maritimum* and *H. stoechas* than *A. cordifolia* growing in the under‐canopy site.

**FIGURE 6:**
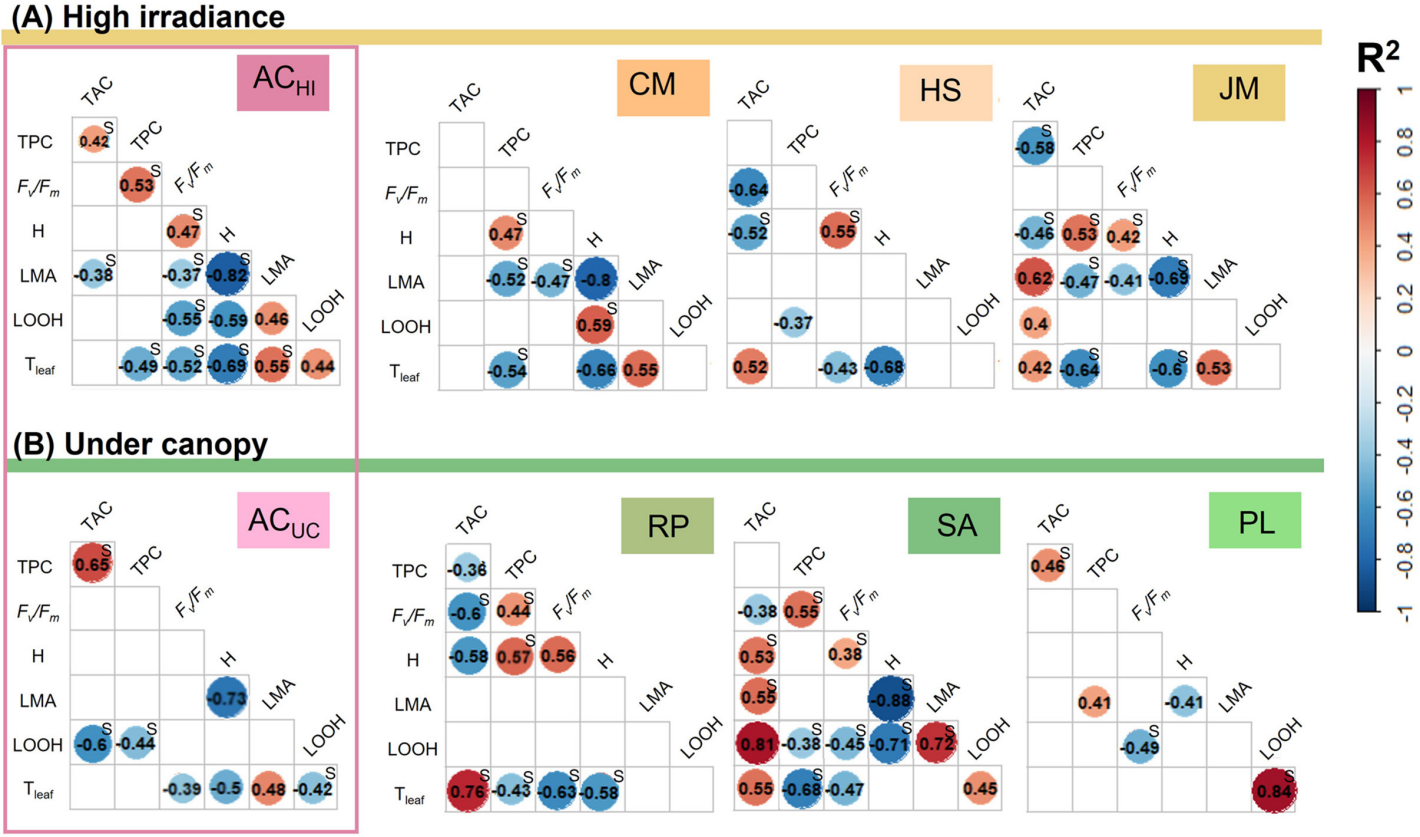
Species significant correlations between physiological parameters of the different species at the high irradiance (HI—yellow) and under canopy (UC—green) sites. Numbers correspond to the highest correlation coefficient (*r* ^2^) found between Pearson or Spearman methods (the latter is indicated with an S). The colour scale from red to blue guides the direction and strength of the significant correlation. Species and traits full names can be found in Tables S1 and S2 respectively.

The species seasonal multidimensional plots (Figure 5B) allowed us to contrast whether species dissimilarity occurred, also at the seasonal level, and if different species shifted towards similar strategies in specific seasons. In summer, almost all species (with the only exception of *R. peregrina* ) shifted towards higher values in the *X* and *Y* MDS axes, that is, towards increased leaf temperature and LOOH and decreased *F*_*v*_/*F*_*m*_, leaf area, angle, and TPC. The high‐irradiance natives and the coexistent *A. cordifolia* showed a common strategy shifting towards lower values on the *X*‐axis in winter. This space corresponds to an increased hydration and LOOH, as well as a decrease in LMA and TAC. Indeed, LMA correlated significantly with leaf temperature for the exotic species *A. cordifolia* at both sites, as well as for the native species *C. matitimum* and *J. maritima* (*r* ^2^ between 0.48 and 0.55, Figure 6). Similarly, in the high irradiance site, all species showed a negative correlation between leaf temperature and hydration (*r* ^2^ within −0.6 and −0.69, Figure 6). TPC also showed a significant negative correlation with leaf temperature in most species (*r* ^2^ between −0.43 and −0.68, Figure 6) except *A. cordifolia* growing under the canopy, *H. stoechas*, and *P. lentiscus* . Meanwhile, *H. stoechas*, *J. maritimum*, *R. peregrina* , and *S. aspera* showed a significant positive correlation between leaf temperature and TAC (*r* ^2^ between 0.42 and 0.76, Figure 6).

## 4 Discussion

The examined native and exotic species demonstrated unique physiological responses to the environmental conditions, illustrating distinctive strategies in both analysed sites. As anticipated, the analysed parameters were influenced by seasonal variations across all species. As described by Ciccarelli et al. (2016), in Mediterranean sea cliff ecosystems, the stressful combination of high irradiance, high temperatures, and low rainfall, typical of the summer season, may have been intensified by the shallow soil which displays a poor water storage capacity. In these ecosystems, winter stress, caused by high solar radiation and low temperatures, has been described to affect multiple Mediterranean species (Morales et al. 2016; Pérez‐Llorca et al. 2019; Muñoz et al. 2021). Nearby, pine groves of *P. halepensis* with undergrowth of sclerophyll shrubs create a distinct microclimatic condition, with reduced light intensity and higher soil water retention. These differential microhabitat conditions determine species responses to multiple stresses across the annual climatic variations (Zandalinas et al. 2018). Despite some shared physiological dynamics being observed (minimum phenol contents during summer, lipid hydroperioxide increases in summer), when responding to those annual climatic variations, the exotic *A. cordifolia* exhibited a markedly differential ecophysiological response when compared to native species thriving under both Mediterranean pines and in cliff ecosystems.

Plasticity in morphological and structural traits is crucial for survival in Mediterranean high irradiance ecosystems, which the exotic *A. cordifolia* might not excel in. In the high irradiance ecosystem during summer, temperatures exceeded 30°C, but the exotic species was the only one to reach leaf values over 40°C. This was accompanied by the lowest *F*_*v*_/*F*_*m*_ values recorded at that site, suggesting that it may have experienced marked thermal stress and impaired physiological performance, in contrast to the native species. Structural changes such as leaf angle variation have been largely proven to be a successful strategy to reduce light absorption in some Mediterranean macchia plants (Werner et al. 1999; Oliveira and Peñuelas 2004; Pérez‐Llorca et al. 2019). For instance, Werner et al. (1999) demonstrated how *Cistus albidus* and *Cistus monspeliensis* revealed a shift in leaf angle from spring to summer that prevented photoinhibition, with a more vertical leaf position in the latter. In our study, all native species growing in high irradiance habitats maintained more vertical leaves across the year compared to the species growing under canopy conditions, thus reducing light absorbance, which contributed to avoiding summer photoinhibition. Another structural modification contributed to preventing summer photoinhibition of all studied species: an increase in leaf dry matter per surface during summer, autumn, and spring. Large LMA is associated with high leaf thickness and density, which appears to be an adaptation to stressful environments like those in the Mediterranean climate (Niinemets 1999). In our study, *C. maritimum* , *J. maritima* , *H. stoechas*, and *P. lentiscus* showed strong accumulation of cell walls and/or osmolytes with LMA values above 120 g m^−2^, the threshold to consider a species a sclerophyte (Flexas et al. 2014), while *A. cordifolia* remained below this value. These thicker leaves might reduce evapotranspiration water loss and thus increase the maximum photosynthetic rate (Wright et al. 2004; Porter et al. 2009; de la Riva et al. 2016). Water accumulation rendered resistance to recurrent dry periods, and despite natives growing in Mediterranean cliffs possessing a high water‐storage capacity, like *C. maritimum* and other *Helichrysum* Mediterranean species (*Helichrysum italicum*; Ciccarelli et al. 2016), *A. cordifolia* exhibited an even higher water content.

The high leaf water content was the main distinctive characteristic of the exotic species. Manipulative experiments with *A. cordifolia* advanced relevant drought stress memory via modifications of the photosystems' antenna and reaction centres, being able to recover from a harsh reduction of the water content reaching 30% of the relative water content (Pintó‐Marijuan et al. 2017). In this way, our study highlights the strong variation also found under natural conditions in leaf water content, doubling from 10 to 20 gH_2_O g^−1^ DW from summer to winter. *A. cordifolia* water content also surpassed another Mediterranean invasive succulent, such as *C. edulis* , with maximum hydration of 10.5 gH_2_O g^−1^ DW in spring (Fenollosa et al. 2017). The strategy to cope with summer stress of *A. cordifolia* was not centered on structure and cell wall accumulation (given the low LMA) to avoid water loss, but on the concentration of compatible osmolytes that reduced osmotic potential and facilitated water uptake from the soil (as supported by highest leaf hydration in this habitat), which are known to protect this species under water stress photoinhibition (Herppich and Peckmann 1997). Besides morphological adaptations, all Mediterranean native species showed an increased non‐phenolic antioxidant capacity in summer compared to winter (except for *P. lentiscus* which sustained high values across seasons), whereas *A. cordifolia* showed a strongly reduced antioxidant capacity in summer at both sites. Minor decreases in photosynthetic efficiency of the invasive species *Carpobrotus aff. acinaciformis* have been observed in open, coastal sites in contrast to the relatively moderate shade of the understories of Mediterranean shrublands and woodlands (Traveset et al. 2008). However, in our study, we found not only minor decreases in *F*_*v*_/*F*_*m*_ but also photoinhibition, revealing a stronger light sensitivity of *A. cordifolia* in contrast with the other natives growing in the open habitat. This increased sensitivity to high light might be due to a higher evaporative demand and different tissue properties of sun‐exposed leaves compared to shallow leaves (Niinemets and Valladares 2004; Díaz‐Barradas et al. 2018).

At the under canopy site, *A. cordifolia* seemed to perform better than in the high irradiance site: (1) not showing a *F*_*v*_/*F*_*m*_ decrease in summer as it did in the high‐irradiance site, (2) not showing a lipid hydroperoxides peak during winter, and (3) maintained leaf temperature values very similar to the ambient temperatures. In this site, the high water content maintained by the exotic species was the main difference from the natives. *A. cordifolia* 's strong water hydration and sustained PSII efficiency was achieved by plastic adjustments of leaf mass area in summer, following a similar strategy to native species growing under high light, and an impressive increase in phenol content during autumn, winter, and spring. At a lower scale, all other species showed either a slight phenol content increase in winter or spring (compared to summer), and/or showed a significant negative correlation between phenol content and temperature, suggesting a common mechanism to face winter stress. Due to their numerous hydroxyl groups, phenolic acids, flavonoids, stilbenoids, and lignans are powerful antioxidants that can scavenge harmful reactive oxygen species (ROS) in plants under various abiotic stressors, including low temperatures (Šamec et al. 2021). Sorce et al. (2019) observed seasonal variations in Mediterranean species such as *Achillea maritima*, *Ammophila arenaria* , and *H. stoechas*, which coincide with our results to describe an increased phenol content when plants were submitted to low temperatures. Additionally, the light‐screening role of phenolic compounds (Šamec et al. 2021) may contribute to photoinhibition avoidance during winter in open areas, by absorbing light in the blue‐green and ultraviolet spectra (Ferreira da Silva et al. 2012; Gould et al. 2018). Specifically, anthocyanins have been described to increase with low temperatures (Nikiforou et al. 2011; Muñoz et al. 2021). This is a consequence of anthocyanin biosynthesis genes being upregulated after low temperatures (Ilk et al. 2015; Schulz et al. 2015; Šamec et al. 2021), and the transition from autumn to winter (shorter days and reduced red/far‐red light ratios) that triggers polyphenol accumulation (Zhang et al. 2016; Wang et al. 2019). This strategy may safeguard photosystems by selectively filtering light and efficiently scavenging oxidative species, thereby averting uncontrolled photodamage. Indeed, only species growing under high irradiance, including *A. cordifolia* , showed an increase in lipid hydroperoxides during winter along with a sudden increase in leaf water content, not accompanied by a decrease in the PSII efficiency. Lipid peroxidation might be triggered by ROS produced as a result of excess excitation energy in chloroplasts under environmental stressors and could cause oxidative damage (Triantaphylidès and Havaux 2009; Noctor et al. 2018). However, when produced transiently, ROS activate cell‐signalling pathways, both inside and outside the chloroplasts, to promote biological processes through antioxidant and redox systems (Foyer and Noctor 2005; Muñoz and Munné‐Bosch 2020). Thus, the mechanisms displayed by the different species were efficient to protect photosystems, and the increase in lipid hydroperoxides might be playing a light‐induced signalling role. Overall, the moderate shade of Mediterranean forests dominated by *P. halepensis* facilitated the inland spreading of *A. cordifolia* by reducing high light stress without exerting strong physiological limitations.

The ecophysiological similarity among natives in each site was higher than with *A. cordifolia* , which could be attributed to the environmental filtering hypothesis (Kraft et al. 2014). Native species growing under canopies or on open cliffs exhibit unique characteristics that enable them to inhabit these specific habitats (Ciccarelli et al. 2016). Notably, while maintaining a distinctive physiological response, *A. cordifolia* exhibited a slightly higher similarity to natives in each community, which reflects environmentally driven plasticity (Nicotra et al. 2010). This similarity was particularly evident in the open habitat, where the exotic species shared a highly similar physiological response with *C. maritimum* and *H. stoechas*, given the multidimensional proximity of AC_HI_ to CM and HS in Figure 5. The slightly elevated physiological convergence in this habitat could be a consequence of more challenging conditions (Flexas et al. 2014; Ciccarelli et al. 2016; Zandalinas et al. 2018). Moreover, Drenovsky et al. (2012) observed a similar response comparing multiple native and invasive grasses and forbs in the Intermountain West (USA), revealing that native and invasive species tend to converge on functionally similar traits when exposed to resource‐poor environments.

The multidimensional analysis revealed that the physiological response of *A. cordifolia* growing under high‐irradiance conditions displayed greater similarity to dominant native species than *A. cordifolia* growing under the canopies with the respective natives. The limiting similarity hypothesis postulates that species require empty niches to succeed in a new community (Weiher and Keddy 1995; Enders et al. 2020; Fagúndez and Lema 2019). High similarity or redundancy may prevent exotics from outcompeting natives (Tordoni et al. 2020). Since differentiation to the native communities enhances invasion success (Kraft et al. 2014; Divíšek et al. 2018; Renault et al. 2022), our results on physiological dissimilarity suggest that *A. cordifolia* might pose a greater risk of invasive success under closed canopies. Its marked physiological plasticity allows it to perform well under low light, in response to high temperatures, and drought conditions expected to become more frequent with climate change. In contrast, native understorey species are typically less equipped to cope with the combined stress of elevated temperatures and reduced water availability. Although *A. cordifolia* 's protective mechanisms are somewhat limited under intense irradiance, its overall resilience suggests that climate change may further enhance its competitive advantage. Management efforts should prioritize under‐canopy habitats near residential areas where *A. cordifolia* continues to be planted, as these locations might be particularly vulnerable to invasion with climate change.

## 5 Conclusions

*A. cordifolia* , a species native to South Africa, that has occupied multiple habitats in Mediterranean‐type ecosystems and which holds an impressive amount of leaf water content, might pose a greater risk of spread under closed canopies, but not in cliff ecosystems, given its high‐light sensitivity in the latter and the higher ecophysiological dissimilarity to native species growing under canopies. According to the limiting similarity hypothesis, a higher dissimilarity to the native community may benefit the alien species establishment due to empty niches, reduced competition, or a lack of natural enemies. The habitat‐specificity and potential success of *A. cordifolia* provide essential information for cost‐efficient biodiversity management resources by prioritizing certain areas.

We employed, for the first time, physiological annual responses to compare non‐native and multiple native species dissimilarities, emphasizing the seasonal variability in morphological and biochemical traits and the intricate nature of plant functioning. This approach enabled us to discern common and distinctive response patterns specific to diverse stressors, underscoring the importance of physiological dynamics beyond mere trait differences when evaluating species dissimilarity. Our perspective aligns with the findings of Fagúndez and Lema (2019), who demonstrated that physiological responses offer valuable insights into the effects of competition. This alignment resonates with the growing interest in the plant metabolome in ecophysiological studies (Walker et al. 2022) and leaf metabolic traits, which have been recognized as a concealed dimension of plant form and function (Walker et al. 2023). Addressing the prevalence or absence of the limiting similarity hypothesis, considering the plant's physiological responses, is crucial for understanding plant evolutionary dynamics, and it can also have positive implications for biodiversity conservation. Indeed, the concept of limiting similarity has been proposed as a management strategy to bolster the resistance of native communities to invasion (Hess et al. 2020) or to enhance overall biodiversity (Cadotte et al. 2021), which in our study we extended to physiological dissimilarity.

## Author Contributions

M.P.‐M., E.F., and S.M.‐B. designed the experiments. M.P.‐M. and E.F. collected the samples, measured the ecophysiological parameters, performed the biochemical analyses, and analyzed the data. E.F. and M.P.‐M. prepared the manuscript with the help of S.M.‐B. All authors revised the final version of the manuscript.

## Conflicts of Interest

The authors declare no conflicts of interest.

## Supporting information


**Data S1:** ppl70455‐sup‐0001‐supinfo.docx.

## Data Availability

The data that support the findings of this study are available from the corresponding author upon reasonable request.

## References

[ppl70455-bib-0001] Abdallah, M. , C. Douthe , and J. Flexas . 2022. “Leaf Morpho‐Physiological Comparison Between Native and Non‐Native Plant Species in a Mediterranean Island.” Biological Invasions 24: 2597–2612.

[ppl70455-bib-0003] Barros, L. 2008. “Antioxidants Activity of *Agaricus sp* Mushrooms by Chemical, Biochemical and Electrochemical Assays.” Food Chemistry 111: 61–66.

[ppl70455-bib-0004] Bou, R. , R. Codony , A. Tres , E. A. Decker , and F. Guardiola . 2008. “Determination of Hydroperoxides in Foods and Biological Samples by the Ferrous Oxidation‐Xylenol Orange Method: A Review of the Factors That Influence the Method's Performance.” Analytical Biochemistry 377: 1–15. 18358821 10.1016/j.ab.2008.02.029

[ppl70455-bib-0005] Buck, A. L. 1981. “New Equations for Computing Vapor Pressure and Enhancement Factor.” Journal of Applied Meteorology 20: 1527–1532.

[ppl70455-bib-0066] Cadotte, M. W. , L. J. Potgieter , C. J. Wang , and J. S. MacIvor . 2021. “Invasion Theory as a Management Tool for Increasing Native Biodiversity in Urban Ecosystems.” Journal of Applied Ecology 58, no. 11: 2394–2403.

[ppl70455-bib-0006] Carmona, C. P. , F. de Bello , N. W. H. Mason , and J. Leps . 2016. “Traits Without Borders: Integrating Functional Diversity Across Scales.” Trends in Ecology & Evolution 31: 382–394. 26924737 10.1016/j.tree.2016.02.003

[ppl70455-bib-0007] Ciccarelli, D. , P. Picciarelli , G. Bedini , and C. Sorce . 2016. “Mediterranean Sea Cliff Plants: Morphological and Physiological Responses to Environmental Conditions.” Journal of Plant Ecology 9: 153–164.

[ppl70455-bib-0008] de la Riva, E. G. , M. Olmo , H. Poorter , J. L. Ubera , and R. Villar . 2016. “Leaf Mass Per Area (LMA) and Its Relationship With Leaf Structure and Anatomy in 34 Mediterranean Woody Species Along a Water Availability Gradient.” PLoS One 11: e0148788. 26867213 10.1371/journal.pone.0148788 PMC4750855

[ppl70455-bib-0009] Díaz‐Barradas, M. C. , M. Zunzunegui , L. Alvarez‐Cansino , M. P. Esquivias , J. Valera , and H. Rodríguez . 2018. “How Do Mediterranean Shrub Species Cope With Shade? Ecophysiological Response to Different Light Intensities.” Plant Biology 20: 296–306. 29125662 10.1111/plb.12661

[ppl70455-bib-0010] Divíšek, J. , M. Chytrý , B. Beckage , et al. 2018. “Similarity of Introduced Plant Species to Native Ones Facilitates Naturalization, but Differences Enhance Invasion Success.” Nature Communications 9: 4631. 10.1038/s41467-018-06995-4 PMC6219509 30401825

[ppl70455-bib-0011] Domènech, R. , and M. Vilà . 2008. “Response of the Invader *Cortaderia selloana* and Two Coexisting Natives to Competition and Water Stress.” Biological Invasions 10: 903–912.

[ppl70455-bib-0012] Drenovsky, R. E. , A. Khasanova , and J. J. James . 2012. “Trait Convergence and Plasticity Among Native and Invasive Species in Resource‐Poor Environments.” American Journal of Botany 99: 629–639. 22434772 10.3732/ajb.1100417

[ppl70455-bib-0013] Enders, M. , F. Havemann , F. Ruland , et al. 2020. “A Conceptual Map of Invasion Biology: Integrating Hypotheses Into a Consensus Network.” Global Ecology and Biogeography 29: 978–991. 34938151 10.1111/geb.13082 PMC8647925

[ppl70455-bib-0014] Fagúndez, J. , and M. A. Lema . 2019. “Competition Experiment of an Invasive Alien Grass and Two Native Species: Are Functionally Similar Species Better Competitors?” Biological Invasions 21: 3619–3631.

[ppl70455-bib-0015] Fenollosa, E. , and S. Munné‐Bosch . 2018. “Photoprotection and Photo‐Oxidative Stress Markers as Useful Tools to Unravel Plant Invasion Success.” In Advances in Plant Ecophysiology Techniques, edited by A. Sánchez‐Moreiras and M. Reigosa , 153–175. Springer.

[ppl70455-bib-0016] Fenollosa, E. , M. Pintó‐Marijuan , and S. Munné‐Bosch . 2017. “Contrasting Phenotypic Plasticity in the Photoprotective Strategies of the Invasive Species *Carpobrotus edulis* and the Coexisting Native Species *Crithmum maritimum* .” Physiologia Plantarum 160: 185–200. 28058723 10.1111/ppl.12542

[ppl70455-bib-0017] Ferreira da Silva, P. , L. Paulo , A. Barbafina , F. Elisei , F. H. Quina , and A. L. Maçanita . 2012. “Photoprotection and the Photophysics of Acylated Anthocyanins.” Chemistry 18: 3736–3744. 22334328 10.1002/chem.201102247

[ppl70455-bib-0018] Flexas, J. , A. Díaz‐Espejo , J. Gago , et al. 2014. “Photosynthetic Limitations in Mediterranean Plants: A Review.” Environmental and Experimental Botany 103: 12–23.

[ppl70455-bib-0019] Foyer, C. H. , and G. Noctor . 2005. “Redox Homeostasis and Antioxidant Signaling: A Metabolic Interface Between Stress Perception and Physiological Responses.” Plant Cell 17: 1866–1875. 15987996 10.1105/tpc.105.033589 PMC1167537

[ppl70455-bib-0020] Gould, K. S. , C. Jay‐Allemand , B. A. Logan , Y. Baissac , and L. P. R. Bidel . 2018. “When Are Foliar Anthocyanins Useful to Plants? Re‐Evaluation of the Photoprotection Hypothesis Using *Arabidopsis thaliana* Mutants That Differ in Anthocyanin Accumulation.” Environmental and Experimental Botany 154: 11–22.

[ppl70455-bib-0021] Herppich, W. B. , and K. Peckmann . 1997. “Responses of Gas Exchange, Photosynthesis, Nocturnal Acid Accumulation and Water Relations of *Aptenia cordifolia* to Short‐Term Drought and Rewatering.” Journal of Plant Physiology 150: 467–474.

[ppl70455-bib-0065] Hess, M. C. M. , E. Buisson , R. Jaunatre , and F. Mesleard . 2020. “Using Limiting Similarity to Enhance Invasion Resistance: Theoretical and Practical Concerns.” Journal of Applied Ecology 57: 559–565.

[ppl70455-bib-0022] Hulme, P. E. 2007. “Phenotypic Plasticity and Plant Invasions: Is It All Jack?” Functional Ecology 22: 3–7.

[ppl70455-bib-0023] Ilk, N. , J. Ding , A. Ihnatowicz , M. Koornneef , and M. Reymond . 2015. “Natural Variation for Anthocyanin Accumulation Under High‐Light and Low‐Temperature Stress Is Attributable to the ENHANCER OF AG‐4 2 (HUA2) Locus in Combination With PRODUCTION OF ANTHOCYANIN PIGMENT1 (PAP1) and PAP2.” New Phytologist 206, no. 1: 422–435. 25425527 10.1111/nph.13177

[ppl70455-bib-0024] IPBES . 2023. Thematic Assessment Report on Invasive Alien Species and Their Control of the Intergovernmental Science‐Policy Platform on Biodiversity and Ecosystem Services. IPBES Secretariat. 10.5281/zenodo.7430682.

[ppl70455-bib-0025] Kraft, N. J. B. , G. M. Crutsinger , E. J. Forrestel , and N. C. Emery . 2014. “Functional Trait Differences and the Outcome of Community Assembly: An Experimental Test With Vernal Pool Annual Plants.” Oikos 123: 1391–1399.

[ppl70455-bib-0026] Leishman, M. R. , V. P. Thomson , and J. Cooke . 2010. “Native and Exotic Invasive Plants Have Fundamentally Similar Carbon Capture Strategies.” Journal of Ecology 98, no. 1: 28–42.

[ppl70455-bib-0027] Mathakutha, R. , C. Steyn , P. C. le Roux , et al. 2019. “Invasive Species Differ in Key Functional Traits From Native and Non‐Invasive Alien Plant Species.” Journal of Vegetation Science 30, no. 5: 994–1006.

[ppl70455-bib-0028] Morales, M. , M. Pintó‐Marijuan , and S. Munné‐Bosch . 2016. “Seasonal, Sex‐ and Plant Size‐Related Effects on Photoinhibition and Photoprotection in the Dioecious Mediterranean Dwarf Palm, *Chamaerops humilis* .” Frontiers in Plant Science 7: 1116. 27516764 10.3389/fpls.2016.01116 PMC4963400

[ppl70455-bib-0029] Munné‐Bosch, S. 2023. “Achieving the Impossible: Prevention and Eradication of Invasive Plants in Mediterranean‐Type Ecosystems.” Trends in Plant Science 29, no. 4: 437–446. 38040553 10.1016/j.tplants.2023.11.007

[ppl70455-bib-0030] Muñoz, P. , A. Cotado , and S. Munné‐Bosch . 2021. “Transient Photoinhibition and Photo‐Oxidative Stress as an Integral Part of Stress Acclimation and Plant Development in a Dioecious Tree Adapted to Mediterranean Ecosystems.” Tree Physiology 41: 1212–1229. 33388772 10.1093/treephys/tpaa177

[ppl70455-bib-0031] Muñoz, P. , and S. Munné‐Bosch . 2020. “Oxylipins in Plastidial Retrograde Signaling.” Redox Biology 37: 101717. 32979794 10.1016/j.redox.2020.101717 PMC7511966

[ppl70455-bib-0032] Nicotra, A. B. , O. K. Atkin , S. P. Bonser , et al. 2010. “Plant Phenotypic Plasticity in a Changing Climate.” Trends in Plant Science 15, no. 12: 684–692. 20970368 10.1016/j.tplants.2010.09.008

[ppl70455-bib-0033] Niinemets, U. 1999. “Components of Leaf Dry Mass Per Area—Thickness and Density—Alter Leaf Photosynthetic Capacity in Reverse Directions in Woody Plants.” New Phytologist 144: 35–47.

[ppl70455-bib-0034] Niinemets, U. , and F. Valladares . 2004. “Photosynthetic Acclimation to Simultaneous and Interacting Environmental Stresses Along Natural Light Gradients: Optimality and Constraints.” Plant Biology 6: 254–268. 15143434 10.1055/s-2004-817881

[ppl70455-bib-0035] Nikiforou, C. , D. Nikolopoulos , and Y. Manetas . 2011. “The Winter‐Red‐Leaf Syndrome in *Pistacia lentiscus*: Evidence That the Anthocyanic Phenotype Suffers From Nitrogen Deficiency, Low Carboxylation Efficiency and High Risk of Photoinhibition.” Journal of Plant Physiology 168: 2184–2187. 21907444 10.1016/j.jplph.2011.07.011

[ppl70455-bib-0036] Noctor, G. , J. P. Reichheld , and C. H. Foyer . 2018. “ROS‐Related Redox Regulation and Signaling in Plants.” Seminars in Cell & Developmental Biology 80: 3–12. 28733165 10.1016/j.semcdb.2017.07.013

[ppl70455-bib-0037] Noto, L. V. , G. Cipolla , A. Francipane , and D. Pumo . 2023. “Climate Change in the Mediterranean Basin (Part I): Induced Alterations on Climate Forcings and Hydrological Processes.” Water Resources Management 37: 2287–2305. 10.1007/s11269-023-03444-w PMC9891896 40478137

[ppl70455-bib-0038] Oliveira, G. , and J. Peñuelas . 2004. “Effects of Winter Cold Stress on Photosynthesis and Photochemical Efficiency of PSII of the Mediterranean *Cistus albidus* L and *Quercus ilex* L.” Plant Ecology 175: 179–191.

[ppl70455-bib-0039] Pérez‐Llorca, M. , A. Casadesús , M. Müller , and S. Munné‐Bosch . 2019. “Leaf Orientation as Part of the Leaf Developmental Program in the Semi‐Deciduous Shrub, *Cistus albidus* L.: Diurnal, Positional, and Photoprotective Effects During Winter.” Frontiers in Plant Science 10: 767. 31275334 10.3389/fpls.2019.00767 PMC6593066

[ppl70455-bib-0040] Pintó‐Marijuan, M. , A. Cotado , E. Fleta‐Soriano , and S. Munné‐Bosch . 2017. “Drought Stress Memory in the Photosynthetic Mechanisms of an Invasive CAM Species, *Aptenia cordifolia* .” Photosynthesis Research 131: 241–253. 27757688 10.1007/s11120-016-0313-3

[ppl70455-bib-0041] Pintó‐Marijuan, M. , and S. Munné‐Bosch . 2013. “Ecophysiology of Invasive Plants: Osmotic Adjustment and Antioxidants.” Trends in Plant Science 18: 660–666. 24001766 10.1016/j.tplants.2013.08.006

[ppl70455-bib-0042] Porter, H. , Ü. Niinemets , L. Poorter , I. Wright , and R. Villar . 2009. “Causes and Consequences of Variation in Leaf Mass Per Area (LMA): A Meta‐Analysis.” New Phytologist 182: 565–588. 19434804 10.1111/j.1469-8137.2009.02830.x

[ppl70455-bib-0043] Renault, D. , M. C. M. Hess , J. Braschi , et al. 2022. “Advancing Biological Invasion Hypothesis Testing Using Functional Diversity Indices.” Science of the Total Environment 834: 155102. 35398434 10.1016/j.scitotenv.2022.155102

[ppl70455-bib-0044] Šamec, D. , E. Karalija , I. Šola , V. Vujčić Bok , and B. Salopek‐Sondi . 2021. “The Role of Polyphenols in Abiotic Stress Response: The Influence of Molecular Structure.” Plants 10: 118. 33430128 10.3390/plants10010118 PMC7827553

[ppl70455-bib-0045] Schulz, E. , T. Tohge , E. Zuther , A. R. Fernie , and D. K. Hincha . 2015. “Natural Variation in Flavonol and Anthocyanin Metabolism During Cold Acclimation in *Arabidopsis thaliana* Accessions.” Plant, Cell and Environment 38: 1658–1672. 10.1111/pce.12518 25689473

[ppl70455-bib-0046] Sheppard, C. S. , M. Carboni , F. Essl , H. Seebens , DivGrass Consortium , and W. Thuiller . 2018. “It Takes One to Know One: Similarity to Resident Alien Species Increases Establishment Success of New Invaders.” Diversity and Distributions 24: 680–691.

[ppl70455-bib-0047] Simpson, A. , E. Sellers , and S. Pagad . 2023. “Global Register of Introduced and Invasive Species – United States (Contiguous) (ver.2.0, 2022).” Invasive Species Specialist Group ISSG. Checklist Dataset. 10.5066/p9kfftod.

[ppl70455-bib-0048] Singleton, V. L. , and J. A. Rossi . 1965. “Colorimetry of Total Phenolics With Phosphomolybdic‐Phosphotungstic Acid Reagent.” American Journal of Enology and Viticulture 16: 144–158.

[ppl70455-bib-0064] Sorce, C. , S. Bottega , and C. Spanò . 2019. “Seasonal and Microclimatic Influences on the Ecophysiology of Mediterranean Coastal Dune Plants.” Estuarine, Coastal and Shelf Science 219: 317–327.

[ppl70455-bib-0049] Tordoni, E. , F. Petruzzellis , A. Nardini , and G. Bacaro . 2020. “Functional Divergence Drives Invasibility of Plant Communities at the Edges of a Resource Availability Gradient.” Diversity 12: 148.

[ppl70455-bib-0050] Traveset, A. , E. Moragues , and F. Valladares . 2008. “Spreading of the Invasive *Carpobrotus aff. acinaciformis* in Mediterranean Ecosystems: The Advantage of Performing in Different Light Environments.” Applied Vegetation Science 11: 45–54.

[ppl70455-bib-0063] Treichel, S. 1975. “Crassulaceensäurestoffwechsel Bei Einem Salztoleranten Vertreter Der Aizoaceae: Aptenia Cordifolia.” Plant Science Letters 4, no. 3: 141–144.

[ppl70455-bib-0051] Triantaphylidès, C. , and M. Havaux . 2009. “Singlet Oxygen in Plants: Production, Detoxification and Signaling.” Trends in Plant Science 14: 219–228. 19303348 10.1016/j.tplants.2009.01.008

[ppl70455-bib-0052] Tuel, A. , and E. A. B. Eltahir . 2020. “Why Is the Mediterranean a Climate Change Hot Spot?” Journal of Climate 33: 5829–5843.

[ppl70455-bib-0053] Valladares, F. , C. C. Bastias , O. Godoy , E. Granda , and A. Escudero . 2015. “Species Coexistence in a Changing World.” Frontiers in Plant Science 6: 866. 26528323 10.3389/fpls.2015.00866 PMC4604266

[ppl70455-bib-0054] Violle, C. , B. J. Enquist , B. J. McGill , et al. 2012. “The Return of the Variance: Intraspecific Variability in Community Ecology.” Trends in Ecology & Evolution 27: 244–252. 22244797 10.1016/j.tree.2011.11.014

[ppl70455-bib-0055] Walker, T. W. N. , J. M. Alexander , P.‐M. Allard , et al. 2022. “Functional Traits 2.0: The Power of the Metabolome for Ecology.” Journal of Ecology 110: 4–20.

[ppl70455-bib-0056] Walker, T. W. N. , F. Schrodt , P. M. Allard , et al. 2023. “Leaf Metabolic Traits Reveal Hidden Dimensions of Plant Form and Function.” Science 9: eadi4029. 10.1126/sciadv.adi4029 PMC10468135 37647404

[ppl70455-bib-0057] Wang, F. , L. Zhang , X. Chen , et al. 2019. “SlHY5 Integrates Temperature, Light, and Hormone Signaling to Balance Plant Growth and Cold Tolerance.” Plant Physiology 179: 749–760. 30563923 10.1104/pp.18.01140 PMC6426432

[ppl70455-bib-0058] Weiher, E. , and P. A. Keddy . 1995. “Assembly Rules, Null Models, and Trait Dispersion—New Questions Front Old Patterns.” Oikos 74: 159–164.

[ppl70455-bib-0059] Werner, C. , O. Correia , and W. Beyschlag . 1999. “Two Different Strategies of Mediterranean Macchia Plants to Avoid Photoinhibitory Damage by Excessive Radiation Levels During Summer Drought.” Acta Oecologica 20: 15–23.

[ppl70455-bib-0060] Wright, I. J. , P. B. Reich , M. Westoby , et al. 2004. “The Worldwide Leaf Economics Spectrum.” Nature 428: 821–827. 15103368 10.1038/nature02403

[ppl70455-bib-0061] Zandalinas, S. I. , R. Mittler , D. Balfagón , V. Arbona , and A. Gómez‐Cadenas . 2018. “Plant Adaptations to the Combination of Drought and High Temperatures.” Physiologia Plantarum 162: 2–12. 28042678 10.1111/ppl.12540

[ppl70455-bib-0062] Zhang, K. M. , J. W. Wang , M. L. Guo , W. L. Du , R. H. Wu , and X. Wang . 2016. “Shortday Signals Are Crucial for the Induction of Anthocyanin Biosynthesis in *Begonia semperflorens* Under Low Temperature Condition.” Journal of Plant Physiology 204: 1–7. 27497739 10.1016/j.jplph.2016.06.021

