# Supplementary material for: Invasion Amid the Shadows: Ecophysiological Dissimilarity and Microhabitat Constraints on an Exotic Succulent in a Mediterranean Ecosystem
Source: Physiol Plant. 2025 Aug 15;177(4):e70455. doi: 10.1111/ppl.70455 (PMC12357129; doi:10.1111/ppl.70455)
Supplement: Supplementary file 1 — Data S1: ppl70455‐sup‐0001‐supinfo.docx. [file PPL-177-e70455-s001.docx]

**SUPPLEMENTARY INFORMATION FROM THE ARTICLE**

**Invasion amidst the shadows: ecophysiological dissimilarity and microhabitat constraints on an exotic succulent in a mediterranean ecosystem**

**Fenollosa, Erola^1,2*^, Munné-Bosch, Sergi^1,2^, Pintó-Marijuan, Marta.^1,2^**

^1^Department of Evolutionary Biology, Ecology and Environmental Sciences, University of Barcelona, Avinguda Diagonal 643, 08028, Barcelona, Spain

^2^Institute of Research in Biodiversity (IRBio-UB), Avinguda Diagonal 643, 08028, Barcelona, Spain

**Correspondence:**

*Corresponding author,

E-mail: [erola.fenollosa@gmail.com](mailto:erola.fenollosa@gmail.com)

**Supplementary Table 1.** Studied species description including the acronym used in the figures and a description.

**Supplementary Table 2.** Studied physiological traits including the acronym used in the figures and a description of their biological significance
